# Multiple Hepatic and Renal Hydatid Cysts Managed with Laparoscopic Surgery

**DOI:** 10.1155/2019/6969232

**Published:** 2019-12-16

**Authors:** Lamia Kouba, Bayan Alsaid, Taisser Almeree, Mazen Allouche, Abdulghani Alshalabi

**Affiliations:** ^1^Faculty of Medicine, Damascus University, Damascus, Syria; ^2^Department of General Surgery, Al-Assad University Hospital, Damascus, Syria; ^3^Department of Radiology, Al-Assad University Hospital, Damascus, Syria; ^4^Department of Urology, Al-Assad University Hospital, Damascus, Syria

## Abstract

Cystic echinococcosis is a parasitic disease caused by *Echinococcus granulosus.* The liver and lungs are the most commonly infected organs. We present the first-of-a-kind case of laparoscopic excision of 8 hydatid cysts, of which seven were in the liver and one was in the kidney of a 40-year-old patient. The patient presented with fatigue and fever and a one-year history of vague abdominal pain. Albendazole was administered before surgical intervention. The postoperative follow-up period was notable for a renal fistula. The patient subsequently underwent CT-guided percutaneous removal of a central hepatic hydatid cyst that was inaccessible using laparoscopic techniques. Cystic echinococcosis is endemic in the Mediterranean region. The growing number of immigrants and refugees from endemic areas could increase the prevalence of the disease in nonendemic countries. Therefore, it is important for physicians worldwide to be familiar with the diagnostic modalities and possible treatment options for hydatid disease.

## 1. Introduction

Cystic echinococcosis (CE) or hydatid disease is a helminthic infection caused by the tapeworm *Echinococcus granulosus.* It commonly affects the liver (60-70%) and the lungs (20-30%), and it rarely affects the kidneys (2-3%) [1, 2]. CE is endemic in the Mediterranean region. Due to the increasing migrant population in nonendemic countries, physicians worldwide should become knowledgeable of this disease's characteristics and cutting-edge management techniques.

Although the laparoscopic approach to excise hydatid cysts is done frequently, it is generally performed when the number of cysts is small (less than three) and it is rarely used to excise cysts presenting in multiple organs like in our case. The particularity of our paper is the successful laparoscopic management of eight hydatid cysts in one patient with seven hepatic cysts and one renal cyst. To our knowledge, this is the first case of its kind reported in the literature.

## 2. Case Presentation

A 40-year-old Caucasian female presented to our center in November 2016 with a one-year history of vague abdominal pain in her right upper quadrant and left flank. Her chief complaint was fatigue and fever for the past ten days.

Her past medical history was notable for multiple hydatid cysts in the liver and lungs with a solitary cyst in the kidney diagnosed three months ago, and since then, she was started on albendazole 400 mg twice daily.

**Figure 1:**
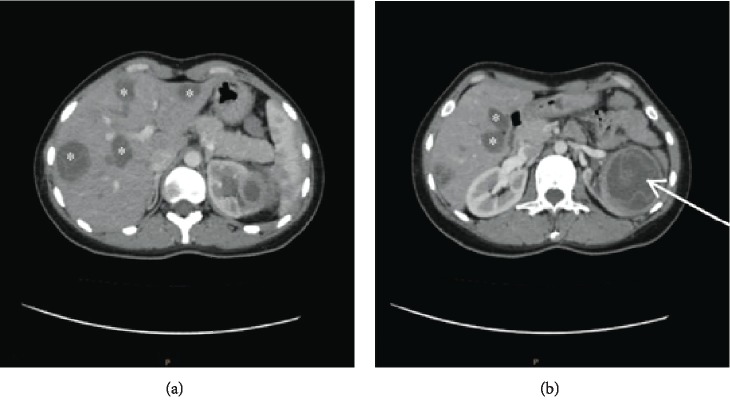
Abdominal CT scan of the patient showing (a) multiple cysts in the liver “∗” and (b) multiple cysts in the liver “∗” and a solitary cyst in the kidney “arrow”.

The patient was admitted to the hospital for reassessment; her clinical examination was remarkable for liver enlargement and left lumbar tenderness. The serologic test for hydatid cysts (indirect hemagglutination test) was positive with a titer of 1 : 16384. Other blood tests were within normal limits except for elevated AST (92 U/L). Her computed tomography (CT) scan revealed (see Figure 1) three cysts with variable diameters (3 cm, 1.3 cm, and 1 cm) in the left lung and eight cysts of variable sizes in the liver; one was centrally located, and the largest one was 4 cm in diameter, in addition to a solitary cyst in the left kidney measuring 6.6 cm in diameter.

The patient was scheduled for laparoscopic surgery to excise the renal hydatid cyst and the reachable hepatic hydatid cysts.

Under general anesthesia, the patient was positioned in the French laparoscopic position, and the operation was performed via four trocars.

Surgical gauzes soaked with hypertonic saline were used to surround the surgical field as a preventive measure to avoid spillage of cystic content and intraperitoneal dissemination.

**Figure 2:**
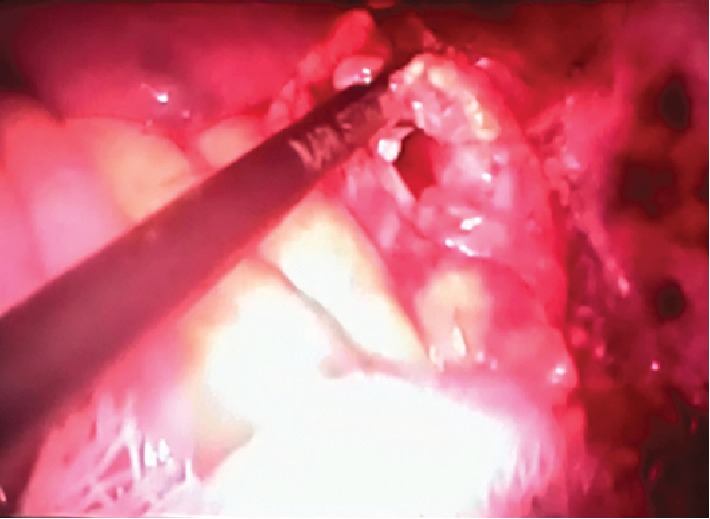
Photo from the surgical monitor depicting an infected hydatid cyst in the left kidney managed laparoscopically.

A large infected hydatid cyst was found in the left kidney (see Figure 2); it was punctured, and the purulent content was aspirated. The remaining cavity was well irrigated, and the entire germinal layer was removed.

In the liver, eight hydatid cysts were found, two in segment II, two in segment IV, one in each of the segments V, VI, and VII, and one intraparenchymal cyst, which was inaccessible by laparoscopic means. Each cyst was punctured, instilled with hypertonic saline for 8-10 minutes, and then evacuated; the puncture was carefully performed to prevent spillage of the cystic contents; the germinal layer was removed for all cysts.

A Foley catheter (25 mL) was inserted in the renal cyst cavity, and three other drains were placed.

In the first week after surgery, a urinary fistula developed in the left kidney and was managed successfully by inserting a Double-J catheter in the left ureter.

The Foley catheter was removed 10 days after discharge when the abdominal ultrasound confirmed the absence of ascites. The Double-J catheter was removed 6 weeks after discharge.

**Figure 3:**
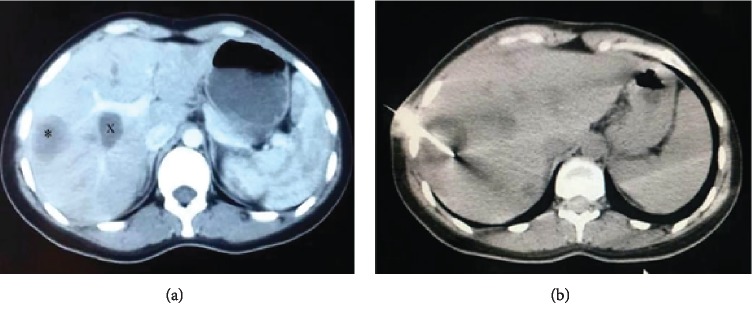
Postoperative abdominal CT scan showing (a) the remaining central hepatic cyst “x” and a remaining cavity “∗”; (b) percutaneous management of the cyst and cavity under CT guidance.

On the eighth-week postoperative follow-up, the central hepatic cyst and a remaining cavity were managed by percutaneous drainage under CT guidance (see Figure 3) using Puncture-Aspiration-Injection-Respiration or PAIR technique.

During the first postoperative year, the patient underwent open thoracic surgery to remove the remaining hydatid cysts in the left lung.

The patient was followed up by serial abdominal ultrasound at regular intervals. No recurrence occurred over 3 years of follow-up.

## 3. Discussion

Despite being an ancient disease, the optimal treatment modality for CE is still debatable. Currently, there are four main treatment modalities for CE: surgery (open or laparoscopic), medical therapy, percutaneous procedures, and finally “watch and wait” strategy for asymptomatic inactive cysts [3].

There is not enough evidence to prove the efficacy of medical treatment alone for hydatid cysts [4]. Broadly, one-third of patients treated with benzimidazole derivatives had recurrence after treatment cessation [5]. Therefore, medical treatment for CE is restricted to patients who are pregnant, have ruptured cysts, or are unable to undergo surgical treatment.

The classical treatment for CE is surgery, and it remains the treatment of choice for complicated cysts, cysts with multiple daughter cysts, those with biliary communications, or those with calcified walls [6]. However, cysts located centrally or adjacent to major vessels render this technique difficult or highly morbid [7].

Following recent advances in technology and the significant morbidity and complications of open surgery, laparoscopic management of hydatid cysts has become a popular alternative. To our knowledge, there are no randomized trials to compare laparoscopic management with other treatment modalities for CE.

According to a systematic review conducted by Dziri et al. [8], laparoscopy is a safe approach to treat hepatic hydatid cysts in selected patients. Nevertheless, there is a need for further assessment of recurrence risk after laparoscopic excision [4].

It was demonstrated that the creation of pneumoperitoneum during laparoscopic surgery could contribute to safer evacuation of the cyst content compared to simple needle aspiration [9]. The laparoscopic approach also displayed superiority to the PAIR technique in terms of leakage control, accessibility, and the capability of inspecting the remaining cavity [9].

However, the PAIR technique was associated with lower mortality and complication rate and higher cure rate than the laparoscopic technique [10].

Furthermore, laparoscopy is rarely used to excise cysts presenting in multiple organs. In a review of world literature on laparoscopic treatment of liver CE, which included 914 patients, only 17 patients had multiple organ involvement and none had liver and kidney involvement like our case [11].

The laparoscopic approach for renal hydatid cyst excision has also been reported in the literature, including transperitoneal and retroperitoneal access. Nephron sparing surgery should be performed whenever possible since CE is a benign condition. Total nephrectomy should be limited to the cases where the kidney is nonfunctioning [12].

In our case, a staged approach was performed to treat the patient's multiorgan cysts. The patient first underwent laparoscopic surgery to remove an infected renal hydatid cyst and seven anteriorly located hepatic cysts. Afterward, we used CT-guided percutaneous drainage for two purposes: to complete the staged approach and remove a posteriorly located hepatic cyst and to treat a remaining cavity. Laparoscopy is preferred over PAIR for superficially located cysts in the anterior side of the liver to avoid spillage of cyst contents in the peritoneum. It also permits a thorough inspection of the remaining cavity to ensure complete eradication of the parasite.

The staged approach enables the curative treatment of difficult cases without resorting to open surgery. This has particular importance in young female patients with multiorgan hydatid cysts [9]. The exclusion criteria for laparoscopic management include deep central cysts, posteriorly located cysts, and calcified cystic walls [13]. It is also not recommended for patients with recurrent disease due to the possibility of excessive intra-abdominal adhesions.

## 4. Conclusion

The laparoscopic approach is a feasible procedure to treat numerous hepatic hydatid cysts (seven in our case) especially when they are located in the periphery and accompanied by extrahepatic hydatid cysts.

A staged approach can be adopted for treating difficult cases to avoid open surgery and its complications.
